# Ethnobotanical survey of plants used as repellents against housefly, *Musca domestica* L. (Diptera: Muscidae) in Budondo Subcounty, Jinja District, Uganda

**DOI:** 10.1186/s13002-018-0235-6

**Published:** 2018-05-10

**Authors:** Kalori Baana, Harriet Angwech, Geoffrey Maxwell Malinga

**Affiliations:** 1 Department of Biology, Faculty of Science, Gulu University, P.O. Box 166, Gulu, Uganda; 2 Department of Environmental and Biological Sciences, University of Eastern Finland, P.O. Box 111, 80101 Joensuu, Finland

**Keywords:** Ethnobotanical knowledge, Insects, Uganda, Repellent plants

## Abstract

**Background:**

The housefly, *Musca domestica* L., is a major public health and domestic pest that spoils food and causes irritation and is a vector of many infectious disease pathogens of medical and veterinary importance. Currently, its control relies largely on chemical pesticides. However, the adverse health and environmental effects of pesticides, risk of development of insect resistance, and bioaccumulation through the food chain emphasize the need to search for environmentally friendly alternatives. This study aimed at documenting traditional knowledge about plants used as repellents against the houseflies by the people of Budondo Subcounty, Uganda.

**Methods:**

An ethnobotanical survey was conducted between November 2016 and June 2017. A total of 372 household members were interviewed on knowledge and use of traditional insect repellents, through face-to-face interviews guided by semi-structured questionnaires administered in nine villages in Budondo Subcounty.

**Results:**

Overall, only 24.5% of the respondents had ample knowledge about insect repellent plants. A chi-square analysis shows a significant association between respondents’ knowledge of insect repellent plants and age, educational status, occupation, religion, and marital status although not with gender. Overall, eight plants from seven families and eight genera were mentioned as repellents. The growth forms encountered were tree, shrub, and herb. Plants that were commonly mentioned by respondents were *Cupressus sempervirens* L. (16.9%), followed by *Lantana camara* L.(16.1%), *Eucalyptus globulus* Labill. (11.0%), *Carica papaya* L. (8.6%), *Cymbopogon citratus* (de Candolle) Stapf (4.3%), *Mentha × piperita* L. (2.4%), *Azadirachta indica* A. Juss (2.2%), and *Ocimum kilimandscharicum* Gürke (0.8%) in descending order. Leaves were the most commonly used plant part (76.9%), followed by the stem/bark (19.8%), flowers (2.2%), and root (1.1%). Burning of the plant materials in order to generate smoke was the most popular method of application.

**Conclusions:**

This study has shown that there are many locally available plants in use by the people of Budondo Subcounty with potency for repelling houseflies. Further studies are needed to identify bioactive compounds responsible for the repellent activity in the different species which could be promoted as sustainable housefly control tools in these remotely located communities of Budondo. Furthermore, studies on the efficacy of these repellent plants or plant parts and their potential toxicological properties should be considered a priority.

## Background

The housefly, *Musca domestica* L. (Diptera: Insecta: Muscidae), is a notorious cosmopolitan pest which causes nuisance and irritation and spoils food and is a vector for many pathogenic organisms that affect humans and livestock [1]. According to Bulter [2], houseflies are vectors because of their coprophagous, indiscriminate, and synanthropic feeding habits. The transfer of pathogens occurs through dislodgement from their hairy body parts and from fly feeding and regurgitation or defecation [3, 4]. Although they do not bite, the fly transmits more than 100 human and non-human animal diseases including bacterial infections like salmonellosis, anthrax, shigellosis, typhoid fever, tuberculosis, cholera and diarrhea, and protozoan infections such as amoebic dysentery [5, 6]. They also transmit eggs of helminths such as pinworms, roundworms, hook worms, and tapeworms as well as viral infections, rickettsial infections, and in some cases, life-threatening *Escherichia coli* [2]. Besides, it is also responsible for transmitting pathogens which cause trachoma and conjunctiva, both of which are estimated to cause approximately 6 million cases of childhood blindness annually worldwide [7]. There are also indications that houseflies are potential carriers of avian influenza flu virus posing threats to humans [8, 9].

However, despite being a major vector for several human and animal diseases, the control aspects of the housefly, *Musca domestica* L., is often neglected [10]. Currently, the control of houseflies relies largely on chemical pesticides. However, the adverse health and environmental effects of pesticides, high cost, risk of development of insect resistance, and bioaccumulation through the food chain [11] emphasize the need to search for low-cost, environmentally friendly alternatives that can complement existing interventions. For developing countries like Uganda whose inhabitants and health and sanitation standards are poor, the use of repellents is the only viable protection against vectors in their contact [12]. In the past few years, many researchers have started exploring the potentials of locally based botanicals against insect pests [12, 13]. Compared to synthetic compounds, plant-based repellents are less toxic [14], less costly, and easily accessible [15], and are still extensively used traditionally as an affordable control option against flies in different communities in Africa [15–17]. For example, in Ethiopia, Karunamoorthi et al. [16] documented nine insect repellent plants belonging to eight genera and families, with the most frequently mentioned being *Boswellia papyrifera* (Del.) Hochst, *Croton macrostachyus* Del., and *Melia azedarach* L. In a rural community of Cameroon, the plant species most commonly used as insect repellents were *Saccharum officinarum* and *Ocimum basilicum* [18]. In Cegere Subcounty, northern Uganda, Anywar et al. [19] reported four plant species: *Ocimum forsskaolii* Benth, *Manihot esculenta* Crantz, *Musa* sp., and *Gossypium hirsutum* L. that are burnt in the house to produce smoke to repel mosquitoes. Unfortunately, however, this indigenous knowledge is being lost as the elderly people die before passing it to younger generations [20]. To ensure the conservation and sustainable utilization of these biological resources, documentation of indigenous knowledge through ethnobotanical studies is urgently needed. So far, studies on the traditional use of ethnobotanical plants as repellents against houseflies and other insects have not have been conducted in Budondo Subcounty. This study was conducted to assess the respondents’ knowledge and to document plants that are traditionally used by the communities of Budondo Subcounty, Jinja District, Uganda, in repelling houseflies/insects.

## Methods

### Study area

This study was conducted in nine villages of Kibibi, Ivunamba, Nawangoma, Buwagi, Kagera, Kyomya, Bufula, Kivubuka, and Buyala B found in Budondo Subcounty, Jinja District, along the northern shores of Lake Victoria, approximately 81 km by road from Kampala (00° 25′ 24 N, 33° 12′4 E). The vegetation consists of grasslands, woodlands, thickets, and bushlands.

### Data collection

**Fig. 1:**
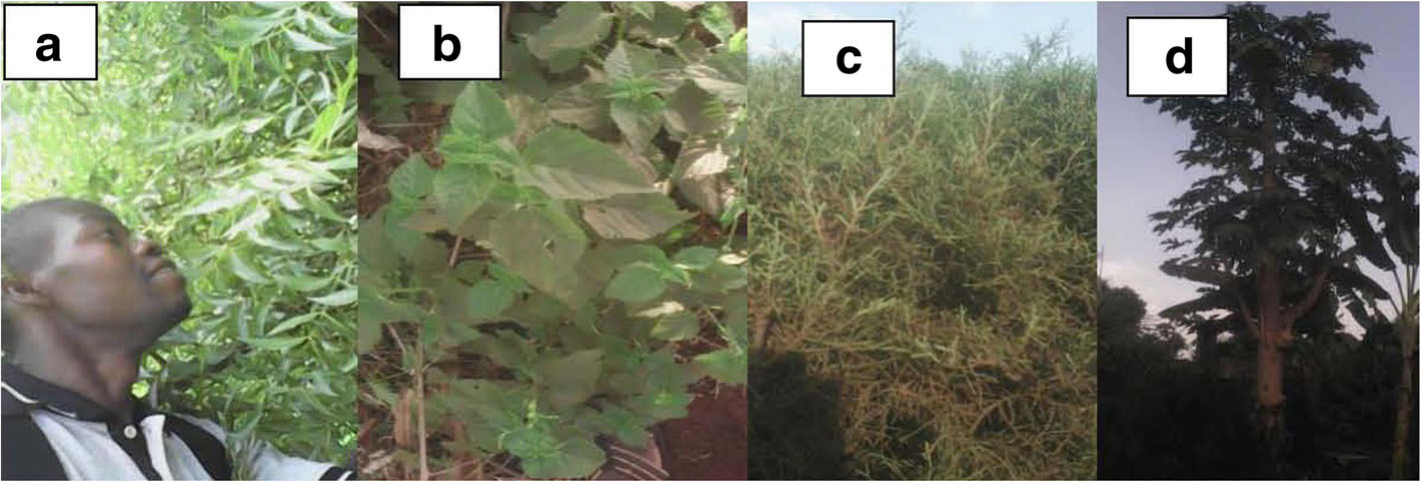
Some of the plants identified as a repellent against housefly, *Musca domestica* L., during a village walk in Nawangoma Village, Budondo subcounty. **a** *Azadirachta indica* A. Juss. **b** *Lantana camara* L. **c** *Cupressus sempervirens* L. **d** *Carica papaya* L.

The study was a cross-sectional descriptive survey and was conducted between November 2016 and June 2017. Data was collected through an ethnobotanical survey employing semi-structured interviews and guided open-ended questionnaires. The households (respondents) for the questionnaires were randomly selected using Krejcie and Morgan table [21]. The questionnaires were administered in Lusoga (the local language in the study area) to 372 local respondents randomly selected using household numbers from nine villages. The household heads who were found at or near their homes at the time of the interview were interviewed, and the researcher did not make return visits to household heads who were not present at the time of administering the questionnaires. This ensured that the individuals who were already interviewed did not influence the views of subsequent respondents. During the interview, the respondents were presented with housefly specimen to guide their responses. The questions asked included the socio-demographic status, the local names of the plants used to repel the fly, part(s) of the plant harvested, and methods of preparation and administration. This was followed by a village walk to verify the plants mentioned and to collect voucher specimen from the plants claimed to have repellent activity (Fig. 1). Key informant interviews and focus group discussions were also conducted to validate the responses obtained from the questionnaires. All the plant materials mentioned by the respondents were identified in the field, and the correctness of the scientific names is checked using the online plant tropicos database [http://www.tropicos.org, accessed 12/06/2017]. Specimens were assigned voucher numbers at the Makerere University Herbarium.

### Data analysis

Descriptive statistics using frequencies and percentages were used to summarize ethnobotanical data in Excel 2010. The association between respondents’ knowledge with their gender, age, educational status, occupation, religion, and marital status was tested with chi-square analysis. Statistical analysis was carried out using SPSS version 23 at 5% level of significance (*P* < 0.05).

## Results

### Socio-demographic characteristics of respondents

The profile of the 372 respondents interviewed in this study is given in Table 1. They comprised 169 (46.5%) males and 203 (53.5%) females and were between the ages of 11 to 91 years (the majority were between 21 to 30 years old, Table 1). Most of the respondents (60.5%) had a basic primary level education. Only 18 (4.8%) of the interviewed respondents were formally employed (Table 1). The majority of respondents belonged to the mainstream religions and were mostly Catholics (26.9%), Muslims (22.3%), Anglican (21.2%), and Pentecostals (20.4%). The majority (66.9%) of respondents were married. Overall, 24.5% (91/372) of the respondents had knowledge about housefly/insect repellent plants (Table 1).

**Table 1:** Socio-demographic characteristics of the respondents (*n = 372*)

Characteristics	Frequency	Percent
Gender		
Male	169	46.5
Female	203	53.5
Age of respondents (years)		
11–20	50	13.4
21–30	143	38.4
31–40	78	20.9
41–50	47	12.6
51–60	24	6.5
Above 60	30	8.1
Educational status		
None	24	6.5
Primary	225	60.5
Ordinary secondary	55	14.8
Advanced secondary	39	10.5
Post-secondary	29	7.8
Occupation		
Peasant (small farmers)	237	63.7
Trader	58	15.6
Civil servant	18	4.8
Student	59	15.9
Religion		
Catholic	100	26.9
Muslim	83	22.3
Anglican	79	21.2
Pentecostal	76	20.4
Traditionalist	7	1.9
Others	27	7.3
Marital status		
Single	123	33.1
Married	249	66.9
Knowledge on insect repellent plants		
Yes	91	24.5
No	281	75.5

### Traditional knowledge on housefly/insect repellent plants

A chi-square analysis shows that there was no significant difference observed in the knowledge of the repellent plants between the gender (*χ*^2^ = 1.274, df = 1, *P* value = 0.256), but there was a significant association with age (*χ*^2^ = 171.2, df = 6, *P* < 0.001), education status (*χ*^2^ = 28.7, df = 5, *P* < 0.001), occupation (*χ*^2^ = 17.2, df = 3, *P* = 0.001), religion (*χ*^2^ = 29.5, df = 5, *P* < 0.001), and marital status of respondents (*χ*^2^ = 35.0, df = 1, *P* < 0.001) (Table 2). The adults were more knowledgeable than the young ones (Table 2). The Catholics were more knowledgeable than the others.

**Table 2:** Knowledge on housefly/insect repellent plants in relation with age, gender, educational status, religion, occupation and marital status of the respondents

Characteristics	Total number of respondents	Knowledge on insect repellent plants		*χ* ^2^	*P* value
		No	Yes		
Gender					
Male	169	123	46	*χ*^2^ = 1.274, df = 1	*P* = 0.256
Female	203	158	45		
Age (years)					
11–20	50	47	3	*χ*^2^ = 171.2, df = 6	*P* < 0.001
21–30	143	136	7		
31–40	78	68	10		
41–50	47	21	26		
51–60	24	4	20		
Above 60	30	5	25		
Educational status					
Illiterate	24	8	16	*χ*^2^ = 28.7, df = 5	*P* < 0.001
Primary	225	174	51		
Ordinary secondary	55	46	9		
Advanced secondary	39	33	6		
Certificate and diploma	13	10	3		
Degree	16	6	10		
Occupation					
Peasant (small farmers)	237	174	63	*χ*^2^ = 17.2, df = 3	*P* = 0.001
Civil servant	18	10	8		
Trader/self-employed	58	41	17		
Student	59	56	3		
Religion					
Catholic	100	66	34	*χ*^2^ = 29.5, df = 5	*P* < 0.001
Muslim	83	62	21		
Anglican	79	62	14		
Pentecostal	76	68	8		
Traditionalist	7	1	6		
Others	27	19	8		
Marital status					
Single	123	116	7	*χ*^2^ = 35.0, df = 1	*P* < 0.001
Married	249	165	84		

### Plants and parts used as repellents

A total of eight plants belonging to eight genera and seven families were reported to be commonly used as a repellent against houseflies in the study area (Table 3). The most commonly represented family was Lamiaceae which had two plants, while the others (Verbenaceae, Myrtaceae, Cupressaceae, Caricaceae, Meliaceae, and Poaceae) consisted of a single species each (Table 3). The most commonly used plant was *Cupressus sempervirens* L. (mentioned by 16.9% of respondents who had knowledge about the plants), followed by *Lantana camara* L. (16.1%), *Eucalyptus globulus* Labill. (11.0%), *Carica papaya* L. (8.6%), *Cymbopogon citratus* (de Candolle) Stapf (4.3%), *Mentha × piperita* L. (2.4%), and *Azadirachta indica* A. Juss (2.2%), and *Ocimum kilimandscharicum* Güerke was the least mentioned (1.1%). The commonest life forms encountered were tree, shrub, and herb. The leaves were the most common plant parts used (76.9%) followed by the stem/bark (19.8%) while the flowers (2.2%) and roots (1.1%) were the least used (Table 3).

**Table 3:** Plants commonly used to repel houseflies and insects in Budondo Subcounty, Uganda

Plant family	Scientific name	English name	Vernacular name (Lusoga)	Voucher number	Part(s) used	Life form	Frequency	Percent	Mode of application
Verbenaceae	*Lantana camara* L.	Tickberry	Kapanga	BK01	Leaf	Shrub	60	16.1	Burn fresh leaves that have been stored in shade for 2 days to generate smoke.
Myrtaceae	*Eucalyptus globulus* Labill.	Eucalyptus	Kalitusi	BK08	Leaf	Tree	41	11.0	Burn fresh leaves after keeping in shade for one night to generate smoke.
Cupressaceae	*Cupressus sempervirens* L*.*	Italian cypress	Krismasi turi, Cedero	BK03	Leaf and stem	Tree	63	16.9	Burning dry (dried in shade for a week) or fresh leaves and stem bark to generate smoke. Fresh leaves are placed in areas where flies are numerous or hanged in the roof and walls of latrines and house to repel the flies
Caricaceae	*Carica papaya* L.	Pawpaw	Omupapali	BK05	Young stem	Tree	32	8.6	Either place or crush fresh young stem in a container to collect the extract/sap and apply on the skin or exposed parts of the body and udder of cows. Burn fresh leaves that have been kept in shade for 2 days to generate the smoke.
Lamiaceae	*Ocimum kilimandscharicum* Güerke	Camphor basil plant		BK07	Leaf and stem	Herb	3	0.8	Burn either fresh, dry (leaves dried under shade for a week), or a mixture of fresh and dry leaves to generate smoke in a house or latrine.
Lamiaceae	*Mentha × piperita* L.	Peppermint	Omujaja	BK02	Leaf, stem, and root	Shrub	9	2.4	Smash fresh leaves/stem/roots, boil to obtain aqueous extract which is sprayed inside the house, or burn fresh leaves, stem, and roots to generate smoke.
Meliaceae	*Azadirachta indica* A. Juss	Neem tree (Eg)	Akarimu	BK06	Flower, leaf, stem, and root	Tree	8	2.2	Burn either fresh, dry, or a mixture of fresh and dry flowers, leaves, stem, and roots to generate smoke or crushing fresh leaves/stem/roots, boil to obtain extract and apply it on the skin/exposed parts of the body.
Poaceae	*Cymbopogon citratus* (de Candolle) Stapf	Lemon grass (Eg)	Kyayi subi	BK04	Leaf and stem	Herb	16	4.3	Burning fresh leaves and stem to generate smoke.

Information on ethnobotanical plant uses was collected through the interviews with the respondents. The plant parts used, life form, and the modes of preparation and administration were also recorded

### Modes of preparation and administration of repellents

The respondents employed a variety of methods to prepare and administer repellent plants (Table 3) The plant materials were used by hanging it in the room while fresh or throwing dry leaves in the garbage collection areas (e.g., *C. sempervirens*); burning fresh or dry leaves, stem, flowers, or root to generate smoke (*L. camara*, *C. sempervirens*, *A.indica*, *C.citratus*, *Metha × piperita*, *O. kilimandscharicum*, and *E. globulus*); crushing fresh parts to obtain extract and applying it on the skin/exposed parts of the body (e.g., for *C.papaya* and *A. indica*, Table 3), and tying them together to form a broom which are used for sweeping away flies from fresh fish and offals on display in the markets (e.g., *C. sempervirens*). Burning or smoldering fresh or dry plant materials in order to generate smoke was the most popular method of application. The plant parts were kept in the shade before they are smoked in the areas where they are required. The places where repellents were used included pit latrines, garbage collection areas, kitchens, fish stores, and markets areas where offal and fresh fish are sold. The respondents reported that the appropriate plant parts were collected when needed either by women or men and that there was no specific dose administered; the amount of plant material used depended on the number of flies in the area and the intensity of odor that each plant produces upon administration. Eight respondents reported that *C. sempervirens* was used to cover offal in the markets to prevent flies from accumulating on it. Two informants reported that they use the sap from young stems of *C. papaya* to spray to their livestock to prevent disturbance from flies and that the same sap is applied on the udder of the lactating cows to serve as a lubricant during milking and to repel flies after milking. During a focus group discussion, ten respondents aged 42 to 70 years mentioned fresh leaves of *L. camara*, *E. globulus*, *C. citratus*, and *C. sempervirens* as being smoked in kitchens during festival seasons to repel the flies that could be attracted by the smell of accumulated meat that has stayed overnight. These plants were also reported to be used during rainy seasons to reduce the dense population of the flies most especially where the food and fruit wastes are deposited.

## Discussion

In the present study, eight plant species were reported to be used as repellents against *Musca domestica* L. by the local inhabitants in the study area. Overall, the number of plants reported in this study was low compared to those obtained in other ethnobotanical studies [16, 17]. Most of the plants reported, e.g., *C.sempervirens*, *E.globulus*, *C.papaya*, *C.citratus*, *M. × piperita*, *A. indica*, and *O. kilimandscharicum*, were not native to Budondo Subcounty, with the exception of *L. camara.* Most of the plants are cultivated at homes except *O. kilimandscharicum* and *L. camara* which are wild. Similar plants have also been previously reported as repellents against traditional insects in several other studies in Kenya [14, 22], Tanzania [23], and Ethiopia [16, 24]. The family Lamiaceae was the most represented insect repellent plant family as found also in an earlier study in Kenya by Kariuki et al. [22]. Lamiaceae is a highly diverse family with a rich source of essential oils which are volatile compounds with a strong odor [25]. The observed insect repellent attributes of these plants might be due to their chemical composition. For example, lemongrass (*C. citratus*), commonly cultivated around homes and whose scent resembles that of lemon, has been shown to contain essential volatile oils that can repel flies. *L. camara* has also been shown to contain a variety of terpenes and alkaloids that can effectively repel flies [26]. Meanwhile, the selection of *E. globulus* may due to its mint-smelling leaves. The bioactive compounds present in these leaves are said to be comparable to the active ingredients present in commercial insect repellents [27].

Our results also showed that only 24.5% of the respondents surveyed had knowledge about the housefly-repellent property of plants. This level of knowledge about insect repellents is poorer than in a study conducted in Ethiopia, in which 97.2% of the respondents had ample knowledge about traditional insect/mosquito repellent plants [16]. This finding is an indication that the indigenous knowledge is rapidly disappearing/eroding from the society. Our results also showed no significant difference in the knowledge of the repellent plants between gender as indicated also in a previous study conducted in Ethiopia with reference to gender and knowledge on insect repellent plants [28]. In contrast, Karunamoorthi and Hailu [24] found no significant association between respondents’ knowledge and gender. These results suggest that the repellent plants are generally known by the members of the community irrespective of their sex. However, the selection of the plants seems to be homogenous among the respondents, indicating that the people of Budondo use almost the same plants to repel the flies. Furthermore, our results also showed a significant association between the respondents’ knowledge on insect repellent plants and their age (Table 2). This can be explained by the fact that elderly people tend to have more knowledge about insect repellents than the young ones because of the frequent exposure to traditional repellent plants [16].

Burning of both fresh or dry leaves and stems to produce in-house smoke to repel the flies was the most favored mode of administration of these plants aligning with the claim of Pålsson and Jaenson [29] in Guinea Bissau which indicated that burning of plants might be effective in repelling flies. Similar results have also been reported in other studies in Kenya [22, 30] and Ethiopia [16] where burning of either fresh or dried repellent plants is one of the common practice to drive away biting insects. It is usually performed using the traditional charcoal stove during the day or in early evenings to repel the flies from dark areas. Although data is scarce on how repellent smokes work, the repellent activity of burned plants might be due to the release of specific volatile compounds (e.g., β-ocimene) created during combustion or from the plant materials themselves [23]. Besides smoking, *C. sempervirens* plants have been used by hanging it on the walls and roofs. In addition, spraying the crushed leaf (*C.papaya*), stem, and roots (*A. indica* and *Metha × piperita*) is also another practice. This finding is consistent with a previous study by Karunamoorthi et al. [16] in Ethiopia which indicates that hanging the leaves of repellent plants in the room and spraying the crushed extracts of repellents plants is a common practice.

Regarding the parts of the plants used for repelling houseflies, the results of the present study indicated that the communities preferentially used leaves, followed by stem/bark, flowers, and roots, and most of the respondents reported the use of fresh leaves. These findings are consistent with those of Youmsi et al. [31] who reported the leaf as the most commonly used repellent plant part. The preference for leaves could be due to the fact that leaves are readily available or that the bioactive compounds or secondary metabolites presumed to be responsible for repelling the flies are more concentrated in the leaves compared to other parts of the plants [31]. Furthermore, the high preference for leaves might result from the strong feeling by the community to preserve these plants. Compared to harvesting plant barks or gathering the whole plant which could cause the extinction of the species, the harvesting of leaves is relatively more sustainable since the plant can regrow new leaves easily, especially during the rainy seasons.

## Conclusion

From this study, it was noted that there are many locally available plants in use by the people of Budondo Subcounty with potency for repelling houseflies. *Cupressus sempervirens*, *Lantana camara*, and *Eucalyptus globulus* were reported as the main repellents against housefly. We recommend further research on these plants to identify the bioactive compounds responsible for the repellent activity in the different species which could be extracted and formulated into useful bioproduct for controlling houseflies. Furthermore, studies on the efficacy of the repellent plants or plant parts and potential toxicological properties should be a priority.
